# New Horizons in Cancer Progression and Metastasis: *Hippo* Signaling Pathway

**DOI:** 10.3390/biomedicines12112552

**Published:** 2024-11-08

**Authors:** Murali R. Kuracha, Uppala Radhakrishna, Sreenaga V. Kuracha, Navyasri Vegi, Jhyama Lhamo Gurung, Benita L. McVicker

**Affiliations:** 1 Department of Internal Medicine, University of Nebraska Medical Center, Omaha, NE 68198, USA; 2 Department of Anesthesiology and Perioperative Medicine, The University of Pittsburgh School of Medicine, Pittsburgh, PA 15213, USA; uppalar@pitt.edu; 3 Comparative Medicine, University of Nebraska Medical Center, Omaha, NE 68198, USA; skuracha@unmc.edu; 4 Shri Vishnu College of Pharmacy, Andhra University, Bhimavaram 534202, Andhra Pradesh, India; navyasrivegi@gmail.com; 5 Department of Pathology, Microbiology, and Immunology, University of Nebraska Medical Center, Omaha, NE 68198, USA; jgurung@unmc.edu; 6 Research Service, Nebraska-Western Iowa Health Care System, Omaha, NE 68105, USA

**Keywords:** *Hippo* pathway, metastasis, mechanotransduction

## Abstract

The *Hippo* pathway is highly evolved to maintain tissue homeostasis in diverse species by regulating cell proliferation, differentiation, and apoptosis. In tumor biology, the *Hippo* pathway is a prime example of signaling molecules involved in cancer progression and metastasis. *Hippo* core elements *LATS1, LATS2, MST1, YAP*, and *TAZ* have critical roles in the maintenance of traditional tissue architecture and cell homeostasis. However, in cancer development, dysregulation of *Hippo* signaling results in tumor progression and the formation secondary cancers. *Hippo* components not only transmit biochemical signals but also act as mediators of mechanotransduction pathways during malignant neoplasm development and metastatic disease. This review confers knowledge of *Hippo* pathway core components and their role in cancer progression and metastasis and highlights the clinical role of *Hippo* pathway in cancer treatment. The *Hippo* signaling pathway and its unresolved mechanisms hold great promise as potential therapeutic targets in the emerging field of metastatic cancer research.

## 1. Introduction

In mammalian physiology, various Receptor Tyrosine Kinase (RTK), Ribosomal S6 Kinase (RSK) signaling pathways, gap junction-mediated intercellular, and other factors influence the maintenance of cell and tissue homeostasis by regulating cell proliferation, growth, and antral development. During the process, various *RTK* signal nodes cross-talk with each other to control cellular integrity and development [1]. Although the complex signaling network mechanism dysregulation confers the aberrant activities of biological functions, the role of tumor-associated micro-environment (TAM) remodeling is yet to be elucidated in metastatic cancer development. The recent discovery of the dynamic role of the *Hippo* Signaling pathway and its association in cell survival can potentially provide the answers for the unresolved questions. This pathway has been widely studied for its significant role in maintaining cellular integrity and homeostasis by controlling cell proliferation, growth, and differentiation. This review provides an opportunity to discuss the *Hippo* pathway and its core kinase elements’ role in cancer progression and metastases. The *Hippo* signaling pathway is a key regulator of organ size, tissue homeostasis, and tumor suppression. It is primarily governed by a series of kinases and transcriptional co-activators, including *MST1/2*, *LATS1/2*, *YAP*, and *TAZ*. These proteins control cellular processes such as proliferation and apoptosis, and their dysregulation is linked to various cancers. The key components of *Hippo* signaling pathway proteins and their functions are summarized in Table 1.

## 2. Hippo Signaling Pathway

The *Hippo/MST* signaling pathway is one of the most attractive research areas in cancer biology. *Hippo* is highly conserved and plays an essential role in cell proliferation, differentiation, migration, maturation, and organ size control in all metazoans [2,3,4,5]. Also, current studies suggested that *Hippo* signaling has also been involved in numerous network connections with receptors, proteins, proteases, and kinases throughout the developmental process to control tissue, organ size, and growth in various organisms. The key components of *Hippo* core kinase cascade consist of a pair of serine/threonine kinases which are *STE20-like protein kinase 1* (*MST1*; known as *STE4*), *MST2* (also known as *STE3*) followed by *Hippo* (*HPO*), and *large tumor suppressor 1* (*LATS1* and *LATS2*), adaptor molecules *Salvador 1* (*SAV1*); and *MOB kinase activator 1A* (*MOB1A* and *MOB1B*) [2,6,7,8,9,10]. During the *Hippo* signaling activation, *Hippo* core kinase elements phosphorylate vital transcriptional coactivators, *Yes-associated protein kinase* (*YAP*), which facilitates the *YAP* degradation and inhibits its nuclear translocalization. However, when *Hippo* signaling is off, *YAP* transcriptionally co-activates with *PDZ-binding motif* (*TAZ*), which is transcriptional enhancer domain proteins and translocate inside the nucleus resulting into cell proliferation to maintain tissue or organ homeostasis.

**Figure 1:**
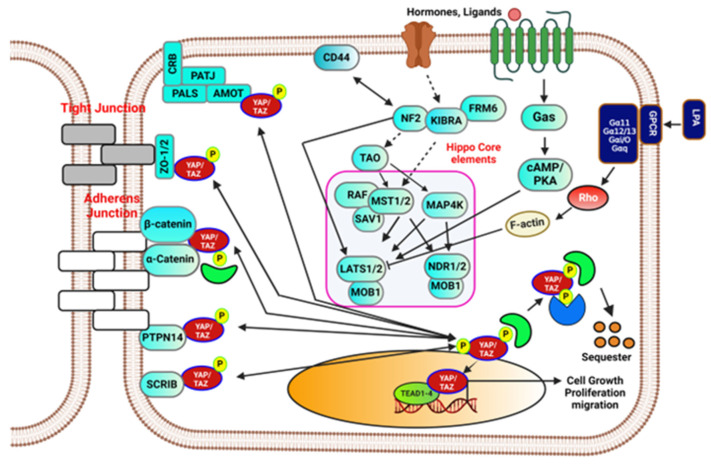
Schematic representation of the general Hippo signaling pathway. The canonical Hippo pathway, activated by GPCR agonists/antagonists and other external stimuli, inhibits cell proliferation and regulates tissue homeostasis. Key upstream components—NF2, MST kinases, SAV1, and MOB adaptor proteins—phosphorylate LATS/NDR. Activated LATS phosphorylates YAP/TAZ, preventing their nuclear entry and promoting 14-3-3-mediated proteasomal degradation in the cytoplasm. Additionally, non-canonical cadherins and tight-junction proteins regulate the Hippo pathway through YAP/TAZ phosphorylation. AMOT, ZO-1/2, and others also phosphorylate YAP to control its cytoplasmic exclusion and activity. Dysregulation of the Hippo pathway leads to YAP-mediated nuclear transcription initiation via TEAD-mediated gene expression.

While the canonical *Hippo* pathway core elements (*MST, LATS*) complex regulates *YAP/TAZ* activity, the *YAP*, *TAZ* and *Yki* complex activity is also controlled by multiple upstream non-canonical signaling. In the canonical pathway, *Hippo* Core elements phosphorylates *YAP/TAZ* complex leading to the *14-3-3* mediated proteasomal degradation in the cytoplasm [11,12]. Studies have shown the presence of numerous upstream molecules which include the *Kibra-Expanded-Merlin* complex in *D. melanogaster* (homologs to *KIBRA*, *WILLIN*, and *NF2* in *mammals*), apicobasal cell polarity (*ABCP*) proteins include *Scribble* (*SCRIB*), *Crumbs* and *Lethal giant larvae*, and *Discs large 1*; *G protein-coupled receptors* (*GPCRs*); and the fourth upstream branch, the large atypical *Cadherins*, *Fat*, and *Dachsous*, triggers *Wts* and *Expanded* (*Ex*) to control tissue growth in *D. melanogaster* [11,13,14]. *Scribble* (*SCRIB*), which might activate core kinase complex [15], and additionally *Crumb’s* complex sequester *YAP/TAZ* proteins in the cytoplasm [16,17,18,19,20]. *G protein-coupled receptors* (*GPCRs*) activate *LATS1* and *LATS2* [21]. Disruption of the *Hippo* signaling constitutively activates transcriptional co-activators *YAP/TAZ*, due to their nuclear localization which interacts with *TEAD* transcription factors (*TEA domain transcription factors*) to promote cell proliferation and oncogenesis [22]. Although mutations in core *Hippo* signaling elements are uncommon, the *Hippo* pathway interacts with various network pathways and molecules, such as *RTK*, *Bone Morphogenic Protein* (*BMP*), *Wnt*, *Notch*, and *Hedgehog*, to control tissue and organ growth across different organisms [23]. Moreover, recent studies have shown that *YAP*, when activated, can promote ferroptosis and lipid peroxide accumulation by upregulating modulators such as *acyl-CoA synthetase long-chain family member 4* (*ACSL4*) and the *lipoxygenase 3* (*ALOXE3*). This suggests that *YAP-ALOXE3* signaling may be a potential biomarker for predicting cancer cell responsiveness to ferroptosis induction, particularly in hepatocellular carcinoma (HCC) [24]. This review offers an opportunity to exploit the upcoming role of the *Hippo* pathway and its core kinases in cancer tissue growth and metastasis. Figure 1 illustrates the *Hippo* signaling pathway.

### 2.1. Hippo Pathway Components

In the canonical *Hippo* signaling pathway, the activation or deactivation of upstream elements *MST1/2* is critical in regulating downstream molecules. *MST1/2* is recruited to the plasma membrane by binding to their regulatory proteins *SAV1/WW45* [25]. Several upstream kinases, Thousand and one Amino acid (*TAO*), *AKT*, and *c-Jun-N terminal Kinase 1* (*JNK1*) phosphorylate *MST1/2* to promote pro-apoptotic signal, including *FOXO3* mediated apoptotic gene expression, and protected from *c-Abl* mediated proteasomal degradation [25,26,27,28,29]. The upstream *Merlin/NF2*, or *Crb3* have enhanced binding efficiency with *LATS1/2* that leads to the plasma membrane recruitment. Then, *MST1/2* and *SAV1* complex phosphorylates *LATS1/2* and *LATS1/2* regulatory subunits of *MOB1A/B* [30]. *LATS1/2* activation is regulated by several *PKA*, *Aurora, and E3 ubiquitin ligases* such as *ITCH, NEDD4, CRL4 (DCAF1), WWP1, and SIAH1/2* [31]. The activated *LATS1/2* and *MOB1A/B* complex triggers the *YAP* and *TAZ* phosphorylation [32].

### 2.2. YAP/TAZ Role

**Figure 2:**
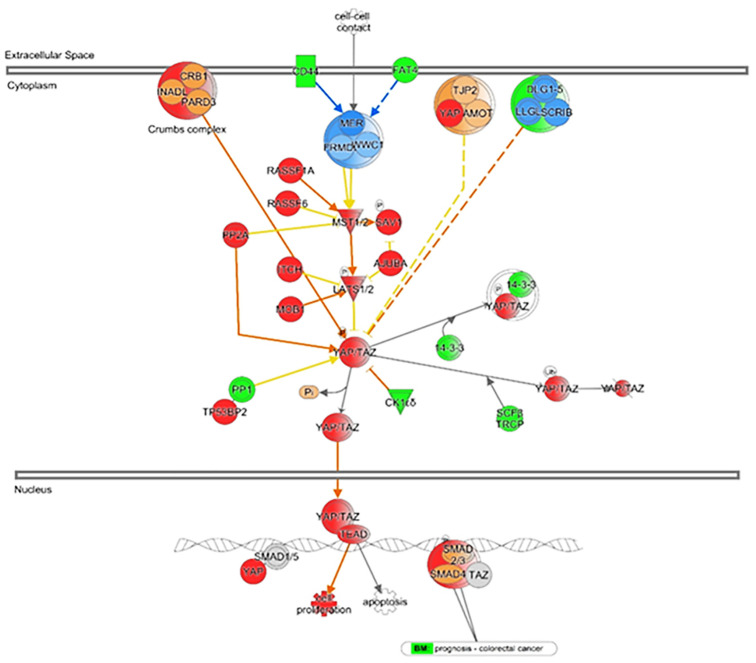
IPA ingenuity pathway analysis: IPA is a software application used for the analysis and interpretation of *YAP’s* complex biological interactions in cancer development. IPA provides a robust platform for exploring *YAP’s* interactions with various biological processes and complex networks. *YAP’s* regulatory mechanisms can develop more targeted therapeutic strategies and advance knowledge of cell signaling pathways.

*YAP/TAZ* are two primary critical effectors of the *Hippo* pathway [32,33]. They form the complex molecule which is found in the cytoplasm and may or may not translocate to the nucleus depending upon the signal. In the canonical Hippo pathway, *LATS1/2* phosphorylation of *YAP/TAZ* facilitates the retention of *YAP/TAZ* complex in the cytoplasm and *14-3-3* mediated proteasomal degradation [32,33]. *YAP* has five serine/threonine residual phosphorylation sites, and *TAZ* has four phosphorylation sites for *LATS1/2* regulation [34]. Mutations in these serine residues resulted in *Hippo* dysregulation, cytoplasmic exclusion of *YAP/TAZ*, and transcriptional activation. The most applicable bonafide residues S127, S381 in *YAP*, and S89, S311 in *TAZ* keep them in a stable state [34]. The phosphate residue of S381 has an additional phosphorylation group by CK1 to form a phosphodegron structure. A critical *SCF E3* ubiquitin *ligase β-transducin repeat-containing protein* (*β-TRCP*) recognizes phosphodegron to regulate *YAP/TAZ* polyubiquitination [35]. It has been seen that nuclear-cytoplasmic shuttling of the endogenous *YAP* is more stable than *TAZ* [36]. Both *YAP/TAZ* have identical and shared features except for the WW domain. *YAP* comprises multi-structural domains, including the TEA DNA-binding domain and two WW1 and WW2 domains [37]. The *TEAD* transcription factors only bind to the first domain, while the WW domain of *YAP/TAZ* (acting as a transcriptional co-activator) binds to the PPxY motif containing transcription factors such as *RUNX, p73, WBP2*, and *ERBB4* [38]. *YAP/TAZ*-induced cell proliferation occurs through the WW domains [37] mediated binding of PPxY motif-containing transcription factors such as *RUNX, p73, WBP2, and ERBB4* [39,40,41,42]. However, *TAZ* has only one WW domain. This variation probably affects transcriptional patterns. The fact that *TAZ^-/-^* mutant mice survive, whereas *YAP^-/-^* mutant mice are embryonic lethal means *YAP* associates with any number of candidate genes to activate transcription [43]. Also, *YAP’s C-terminal PDZ binding motif* is critical in regulating *YAP* activity. *ZO2* interacts with *YAP* in a PDZ-dependent manner, and it has been shown to colocalize with *YAP* into the nucleus [44,45]. The *SRC* homolog of the SH3 binding domain allows binding through *p53 binding protein 2* with the association of WW domain [46]. Based on the IPA network data analysis, which reveals the activity of nuclear *YAP*-associated transcriptional genes (Figure 2).

Besides having numerous molecular events to regulate *YAP/TAZ* expression, localization, and function, several other non-canonical *Hippo* kinases (e.g., *SET7*, *Glycogen synthase kinase 3β* (*GSK-3β*), *CDK1, HIPK2, AKT, JNK*, ubiquitin enzymes, and transcription factors) can also significantly modify *YAP/TAZ* activity. For instance, the transcription factors *SOX2* and *Est-related* genes are direct targets of *YAP* transcription. However, *Fbxw7 E3* ubiquitin is known to reduce *YAP* activity by proteasomal degradation [47]. Further, studies have confirmed that the tyrosine phosphorylation in *YAP* depends on the functional necessity. Both *YES1* and *c-Abl* kinases phosphorylate at tyrosine residue (Y357). *YES1*-mediated tyrosine phosphorylation in *YAP* facilitates translocation of *YAP* inside the nucleus whereas *c-Abl*-induced phosphorylation at Y357 reduces *YAP-TEAD* transcriptional activity [47,48]. *SET7* induces *YAP* methylation, another regulatory mechanism that inhibits the *YAP* cytoplasmic exclusion [49]. Moreover, *LATS*-independent *YAP* phosphorylation is reversed by activating phosphatases, such as *PP1, PP1A*, which significantly rebounds the *YAP/TAZ* activity [50,51]. *PTPN14* is another *YAP* target, which forms a *PTPN14/YAP* complex, and helps to retain *YAP* to the cytoplasm [52,53,54]. Figure 2 represents the IPA network analysis revealing the nuclear *YAP* association.

### 2.3. Nuclear YAP/TAZ Complex

The proof of concept is that the nuclear *YAP/TAZ* is acting as a transcriptional co-activator to enhance the transcriptional gene activity. Studies have determined that *YAP/TAZ* complex binding with *RUNX* or *TEAD* transcription factors increases luciferase overexpression in mammalian cells [55]. Also, *GAL4* DNA binding domain fusion with *YAP/TAZ* provides classical evidence that *YAP* or *TAZ* proteins have a potential C-terminal binding domain to boost the gene’s transcriptional activity [38].

**Figure 3:**
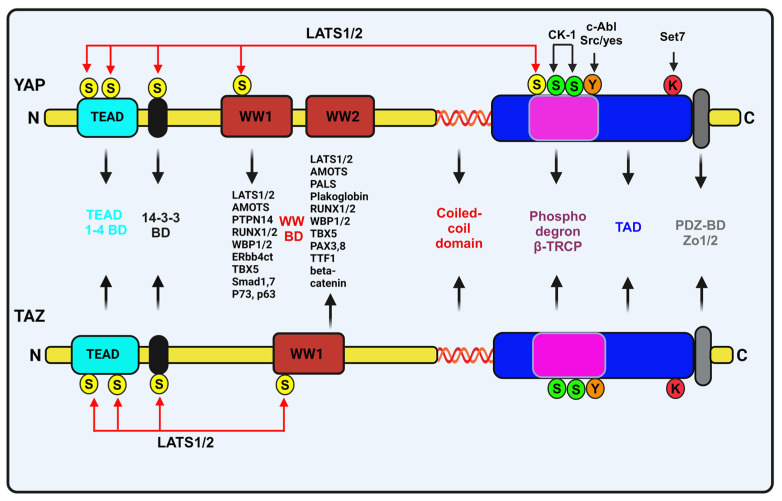
Schematic representation of the structural domains of YAP and TAZ and their interactions with other proteins. LATS1/2 mediated phosphorylation through five serine residues on YAP and four on TAZ highlighted in yellow. The TEAD gene binding domain (TEAD-BD), and 14-3-3 protein binding domain (14-3-3-BD), are indicated, reflecting phosphorylation by LATS1/2. On both proteins, CK-1 phosphorylation sites are shown in green. Set7 methylase targeted c-terminal lysine residue on both YAP/TAZ are marked in red. The transcriptionally active domain (TAD) is located at the c-terminal region, while the PDZ binding domain (PDZ BD) is a small c-terminal domain that interact with PDZ containing proteins. C-Abl phosphorylation at the TAD domain tyrosine residue on both YAP/TAZ is shown in orange.

Several studies have shown that *TEADs* are the main factors interacting with nuclear *YAP* and *TAZ* to regulate the number of cellular functions in mammalian cells [55,56,57,58]. However, any point mutation in the critical amino acid residues at the N-terminal of *YAP*, which is also a *TEAD* binding domain, can remarkably influence *YAP/TAZ* dependent growth and epithelial stem cell growth polarization in soft agar [42,56,58,59,60]. Indeed, patients with Sveinsson’s disease frequently present with retinal degeneration due to a point mutation, (Y421H) in *YAP*, that prevents the *YAP-TEAD* binding interaction [61,62]. Similarly, *TEAD* loss of function phenotype reflects the similarities with *YAP* loss of function phenotypes [59,61,62,63,64]. Thus, *YAP/TEAD* interactions are found to be very critical in maintaining tissue growth and homeostasis. Remarkably, several proteins such as *BIRC5*, *AXL, CYR61, ANKRD1, InhA, CTGF*, and *Col8a1* interact with *YAP* to preserve their activity in vitro, as well as in vivo [15,65,66,67]. However, such investigations relied on *YAP/TAZ* overexpression, potentially highlighting only a specific fraction of *YAP/TAZ* targets. A typical downstream transducer of *TGF-β* and *BMP* pathway *Smad* proteins have been steadily associated with *YAP/TAZ* to regulate tissue homeostasis [20,68]. Additionally, *vestigial-like protein-*4 (*VGLL4*) has been shown to suppress *YAP1/TEAD*-dependent signaling in epidermal squamous cell carcinoma, reducing tumor formation and EMT by inhibiting *YAP1*-dependent transcription and target gene expression [69,70]. Figure 3 illustrates the multiple domains of YAP/TAZ interactions with other proteins. 

### 2.4. YAP Regulation

Canonical and non-canonical mechanisms regulate *YAP* activity in the cytoplasm and maintain the *Hippo* pathway. However, several factors regulate *YAP* activity in a precise manner including the involvement of *G-protein coupled receptors* (*GPCRs*). *GPCRs* comprise a massive family of membrane receptors of three subunits, α, β, and γ. The α subtypes of *GPCRs* (*Gα11, Gα12, Gα13, Gαi, Gαo,* and *Gαq*) have been shown to enhance *YAP/TAZ* activity, whereas *Gαs* reduces nuclear *YAP/TAZ* activity [71]. *GPCRs* regulate *YAP* activity through *Rho GTPase*-mediated F-actin polymerization, which can block *LATS1/2* phosphorylation [71]. One study suggests that the agonists of *GPCRs,* such as *lipoprotein*, *sphingosine-L-phosphate*, *glucagon* and *epinephrine,* can stimulate cell proliferation through *Hippo* signaling [72]. Additionally, *Wnt* signaling can also regulate *YAP* activity by disrupting *β-catenin YAP* complexes facilitating *YAP* relocalization to the nucleus [73]. 

The non-coding endogenous 18–24 nucleotides in length sequences (*miRNAs*) have profound effects in vivo and in vitro gene regulation. Recent studies have noted the increased role of *miRNAs* in many cancers as tumor suppressor molecules. However, *miRNAs* can also have a profound oncogenic role. For example, *miRNA-31* has been found to repress *Hippo* pathway activity leading to poorer outcomes in endometrial cancer patients. In that case, *miRNA-31* acts as an oncogene by binding to the 3′ untranslated region (UTR) of *LATS1/2* which releases *YAP* from the cytoplasm leading to *YAP* nuclear translocation, *cyclin D1* expression, and tumor cell proliferation [74].

*Long non-coding RNAs* (*LncRNAs*) are a highly heterogeneous class of non-coding RNAs typically consisting of more than 200 nucleotides base pairs. Recently, it has been recognized that the aberrant expression of *LncRNAs* has a role in the occurrence and development of different cancers [75,76,77,78]. *LncRNAs* regulate the *Hippo/YAP1* pathway in various cancers. For example, He et al., reported that *LncRNA* expression is elevated in breast cancer which is mediated through the *LncRNA-NF2 axis* [75]. Similarly, higher expression of *LINC02159* was found in non-small cell lung cancer (NCLC) signifying its vital role in the pathogenesis of NCLC growth and metastasis through *Hippo* and *β-catenin* pathways [76]. Thus, *LncRNAs* could be a new potential target molecule that can be used as biomarkers for diagnosing and staging of various cancers.

### 2.5. Role of the Apical Protein Crumbs and AMOT

As noted previously, studies indicate that transmembrane proteins such as *Scribble* and *Crumb* family proteins can have an impinged effect on *YAP/TAZ* activity. As reported, *Crumb* proteins and their partner factors (*PALS* and *PATJ*) combine to form an apical *crumb complex* (*CRB*). The *CRB* binds with *YAP/TAZ* for the maintenance of cytoplasmic localization [20]. The mechanism behind *CRB*-mediated *Hippo* signaling regulation is still unclear; however, *Crumbs* complex has a potential role in regulating *YAP/TAZ* localization in the cell with defects in the *CRB* complex associating with cancer development.

A novel pathway of *YAP* regulation by angiomotin (*AMOT*) has been identified. *AMOTs* have been characterized as *YAP* inhibitors with reports supporting a role of *AMOTs* in the promotion of *YAP* sequestration [73,74,79] or as an activator of the *Hippo* pathway [80,81]. Several mass-spectrometry studies identified *AMOTs* as a binding partner of *LATS* and *YAP/TAZ* complexes [20,82,83,84,85]. Moreover, phosphorylated *LATS* regulates *AMOTs* and *F-actin* interactions [86]. Further, an observed early embryonic phenotype in *AMOT* and *AMOTL2* mutant mice suggested that *AMOTs* act as a *YAP* inhibitor during *Hippo* pathway activation [81,87]. Further, *AMOTs* can behave as a negative modulator to inhibit *YAP* activity in the cytoplasm, and simultaneously as a co-factor to initiate gene activation in the nucleus.

## 3. Hippo’s Role in Epithelial-to-Mesenchymal Transition

Another role of *Hippo* signaling in cancer relates to the apicobasal polarity properties of the *Scribble* complex in the regulation of *YAP/TAZ* activity during *YAP/TAZ* regulation. *Scribble’s* adaptor molecule becomes a *Hippo* kinase activator at the cell membrane [88]. Scribble associates with *Hippo* kinases by forming a complex containing *MST, LATS,* and *TAZ*, which are required for *MST*-mediated *LATS* activation [89]. Loss of epithelial polarity represents the first event of the epithelial-to-mesenchymal transition (EMT). The loss of cell polarity triggers *Scribble* delocalization and inactivation of the *Hippo* signaling pathway causing *YAP/TAZ* complex nuclear translocation. As a result, transcription of target genes leads to the increased expression of mesenchymal markers (e.g., *vimentin* and *N-cadherin*) along with a decline of epithelial markers like *E-cadherin* [13]. During cancer development, *Scribble* inactivation or delocalization from the plasma membrane is common, indicating a role for cell polarity during tumorigenesis. In support, the lack of *Scribble* in mutant mice relates to the presence of highly metastatic tumors [88,90,91]. Moreover, studies demonstrated that the tumor suppressor *LKB1* stabilizes *Scribble* and *Hippo* complex [92]. The stabilization of the complex is mediated by *LKB1* targeting *Par1/MARKs*. These results suggest that the *Scribble/Hippo axis* could represent a main epigenetic event for *YAP/TAZ* regulation in human tumors.

### YAP’s Role in Gynecological Cancers

Studies have noted that *YAP/TAZ* is essential in tumor initiation and progression with *YAP/TAZ* having multiple roles in cancer cell metastasis and drug resistance [93,94]. *YAP/TAZ* activation is also involved in tumor microenvironment (TME), including inflammation, genetic mutations, and Mechanotransduction. A striking role of *YAP/TAZ* has been determined for ovarian cancer, the fifth leading cancer death for women in the USA. Standard treatment options such as surgery and chemotherapy remain inefficient for ovarian cancer indicating the significant need to identify targetable mechanisms. Recent observations suggest that *YAP* elevation leads to transcriptional activity of *TEAD4* gene expression which is associated with a poorer response to the standard treatments in ovarian cancer patients [95]. Overall, the phosphorylation status of *YAP* is important in ovarian cancer survival since the inactivation of *Hippo* signaling leads to *YAP* dephosphorylation and translocation to the nucleus for transcription factor interactions and subsequent cancer cell proliferation [96]. Conversely, the phospho form of the *YAP* signature contributes to positive outcomes with taxane-based adjuvant chemotherapy in ovarian cancer patients [97].

Elevated levels of serological glycoproteins and phospholipids initiate ovarian cancer, such as *lysophosphatidic acid* (*LPA*), one of the most abundant serum *lipids*. *LPA* acts as a ligand that binds to the *GPCRs*, promoting tumorigenesis and metastasis in ovarian cancer. Remarkably, *LPA*-induced *TAZ* transcription leads to enhanced cell migration ability in R182 human ovarian cancer cells [98]. *LPA* induced *YAP/TAZ* dephosphorylation enhances the interaction between *YAP* and *TEAD* to promote ferroptosis in ovarian cancer, making it a promising target for cancer therapy [69]. Additionally, *MIR-129-5p* is extensively involved in regulating *YAP/TAZ* transcriptional activity, consequently leading to the inactivation of *TEAD*-mediated and *Hippo*-dependent gene downregulation. The suppressor role of *MIR-129-5p* in ovarian cancer is associated with poorer survival in ovarian cancer patients [79]. In contrast, *MiR-146a-5p* within extracellular vesicles (EVs) accelerates cervical cancer metastasis by activating the WWC2-mediated *YAP* signaling pathway that enhances EMT [99]. Dysregulation of the *Hippo* pathway is vital in tumor initiation and progression in ovarian cancers. The *YAP*-mediated positive feedback activation loop enhances the *ERBB* signaling pathway to regulate the tumor initiation and progression in ovarian cancer [100]. Moreover, TME induced *fibroblast growth factor* (*FGF*) and the *Hippo* signaling pathway regulate the autocrine/paracrine positive feedback loop which drives the progression of the high-grade serous carcinoma associated with fallopian tube secretory epithelial-derived cells [101]. Additionally, blocking cellular interactions with an RGDKGE-containing collagen fragment enhances the phosphorylation of *LATS1* and *YAP*. Targeting RGDKGE collagen proteins was shown to inhibit ovarian cancer growth, suggesting a novel therapeutic target for ovarian tumor treatment [102,103]. Overall, the *Hippo* signaling pathway plays a pivotal role in gynecological tissue homeostasis in women.

## 4. The Hippo Pathway in Cell Migration and Cancer Metastasis

Cell signaling molecules and their transcriptional factors actively induce numerous cell-autonomous functions such as proliferation, cell plasticity, chemoresistance, and metastasis [94]. The signaling molecules, however, reside in the TME which is a complex environment comprised of cancer cells, stromal cells, cancer-associated fibroblasts (CAFs), extracellular matrix (ECM) components, and immune cells [104]. During cancer cell dissemination, destruction of apicobasal polarity and cytoskeletal network rearrangements are hallmarks of EMT and tumor progression [105]. Several studies have reported that the activation of *Hippo* signal molecules *YAP/TAZ* due to dysregulation of *MST1/2* or *LATS1/2* leads to cancer metastasis [104,105,106]. Similarly, *YAP/TAZ* loss of function studies have reported the rescue of mesenchymal morphology to epithelial morphology [107,108]. Surprisingly, the *YAP1* transcriptional co-activator promotes the growth of mutant *KRAS*-dependent colon cancer cells upon attenuation of *KRAS* allele. Also, *YAP*-mediated signaling is has been implicated in *KRAS*-driven murine lung cancer models [101]. Interestingly, *YAP*-associated *FOS* transcriptional factor is involved in EMT-induced gene regulation where downregulation of junctional proteins like *E-cadherin* and *occludin* was observed [109]. Moreover, *YAP* signaling regulates EMT-induced transcription factors such as *Snail1/2, Slug, ZEB1* (*Zinc Finger E-box-binding Homeobox 1*), and Twist in various cancers [104,110]. These studies suggest that the *Hippo* pathway activates the EMT program in cancer metastasis.

TME crosstalk also plays a key role in regulating cancer cell motility and invasiveness through the action of various cellular biomechanics such as focal adhesion, membrane remodeling, actin protrusion, and cell motility signaling pathways [111]. However, *YAP*-mediated *F-actin* focal adhesion has been revealed to be a crucial determinant of cell migration and plays an important role in promoting tumor cell invasion [104,107,111]. The overexpression of *YAP* causes cytoskeletal rearrangements by promoting *ARHGAP29* to suppress the *RhoA-LIMK-cofilin* pathway, leading to the changes in the dynamics of *F-actin/G-actin* turnover and migration in gastric cancer cells [112]. During this process, *YAP* receives signals from the surrounding stroma which activates *ARHGAP29* to regulate actin dynamics resulting protrusions and metastatic behavior of the tumor cells. Moreover, *ARHGAP29* RNA levels can be elevated in circulating gastric tumor cells [112]. Altogether, these data show that *YAP* provides a clear insight into promoting cancer cell motility by altering the actin dynamics. Another study has identified *YAP* as a fluid mechanosensor in the regulation of genes involved in cancer metastasis. In this scenario, frictional force in the lymphatics activates *YAP* to promote tumor cell migration [113]. Also, the activation of *YAP* in tumor cell motility was shown to involve the action of platelet-derived growth factor (*PDGF*) which mediated *YAP* dephosphorylation and transactivation via the *RhoA/PP-1* cascade in the promotion of pancreatic cancer [114].

**Figure 4:**
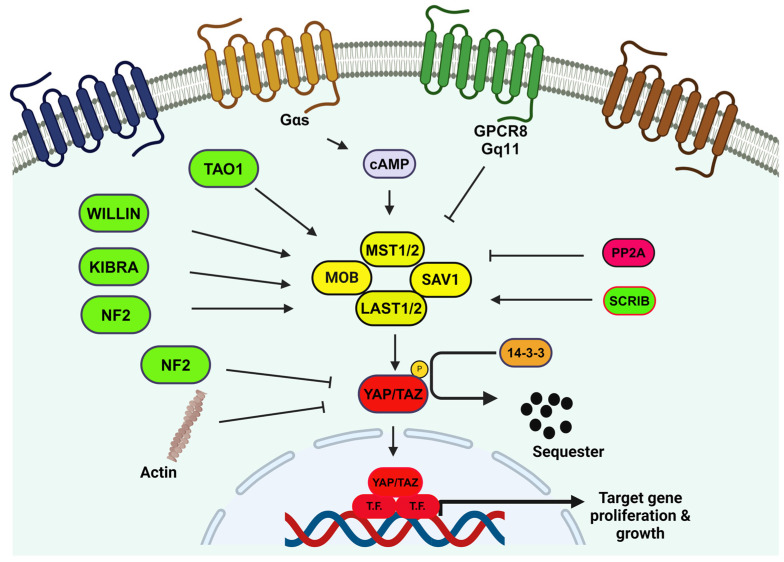
Schematic representation of the *Hippo* pathway. Oncoproteins are highlighted in red, tumor suppressors in yellow, and upstream activators in green. The *LATS1/2* complex phosphorylates *YAP* and *TAZ*, leading to their degradation through binding to *14-3-3* proteins. In both *Drosophila* and *mammals*, *YAP* (*Yes-associated protein*) is regulated by an upstream core kinase cascade that includes *MST1* (also known as *STE4*) and *MST2* (*homologous to Drosophila Hippo [HPO]*), along with large tumor suppressor 1 (*LATS1*) and *LATS2*. Adaptor proteins, such as *Salvador 1 (SAV1)* and *MOB kinase activator 1A/B* (*MOB1A/B*), also play crucial roles in this signaling pathway. The *Hippo* pathway maintains tissue and organ homeostasis by modulating a serine/threonine kinase cascade that phosphorylates the transcriptional coactivators *YAP* and *TAZ*, preventing their nuclear localization and transcriptional activation. This phosphorylation leads to 14-3-3 mediated sequestration and proteasomal degradation of *YAP/TAZ* in the cytoplasm, thereby controlling tissue growth. Key regulatory molecules include *AMOT* family proteins (*angiomotin*), *PP2A* (*protein phosphatase 2A*), *NF2* (*neurofibromin 2*), *KIBRA* (*kidney and brain protein*), *TAO1* (*thousand and one amino acid protein*), and *SCRIB* (*Scribble*).

Other investigations into mechanisms of tumor cell migration led to the study of small *GTPase* family members (i.e., *Rap1*) in Hippo signaling. An independent study that screened for *Rap1* (*Ras-related protein 1*) identified the *Hippo* pathway as a binding partner to the active form of *Rap1* [115]. The *Rap1-Hippo* interaction impeded Hippo autophosphorylation and activation resulting in the suppression of *F-actin* and ultimately the generation of cellular protrusions and cell migration. *Rap1* is known to regulate junction formation and integrin signaling to promote tumorigenesis in various cancers under Ras hyperactivity. Interestingly, *Rap2*, a close homolog of *Rap1*, is antagonized by *Rap1* in endothelial cells and it serves as a mechanosensor to relay stress from ECM, as well as activating *Lats1/2* in response to low stiffness [116]. However, further studies are needed to fully characterize the role of *Rap1* in suppressing *Hippo* signaling activity in human cancers. Overall, Figure 4 summarizes this discussion by highlighting oncogenic protein targets that are regulated through nuclear *YAP/TAZ* co-activation in cancer progression and metastasis.

## 5. Role of Hippo Signaling in the Mechanotransduction

**Figure 5:**
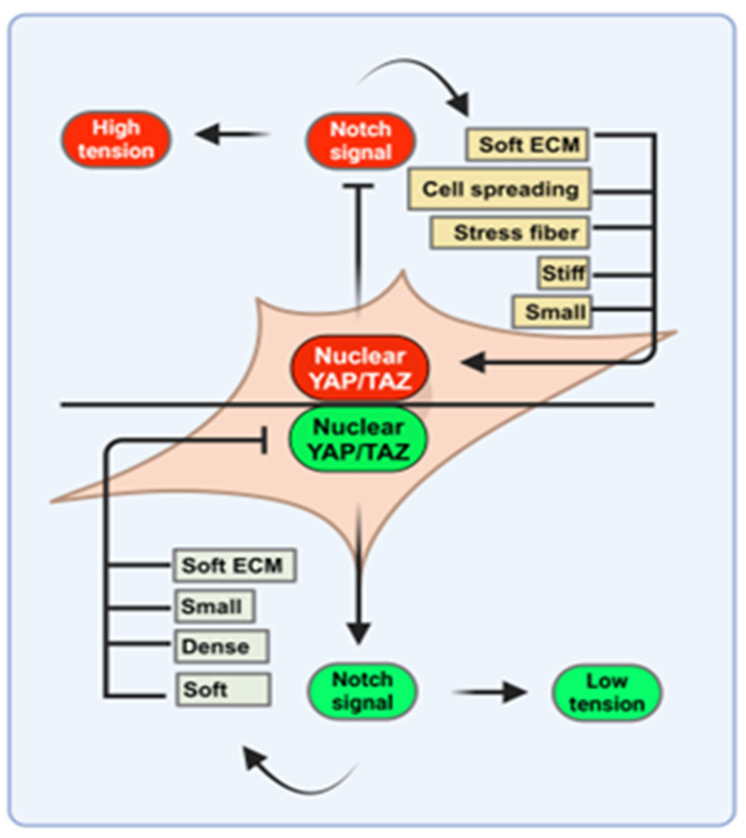
*YAP/TAZ*-mediated mechanical signal regulation. *YAP/TAZ* nuclear transcription can have a potential biological effect by controlling mechanical signal. Activated *YAP/TAZ* preserves cells in an undifferentiated state, consequently stretching in shape, adhesion on rigid surfaces, and low cell density. Oppositely, inactivated *YAP* influences low mechanical signal and cell differentiation.

Mechanotransduction is an emerging field in cell physiology to understand how physical and mechanical forces influence cellular proliferation and differentiation properties to maintain tissue or organ homeostasis. *Hippo*-mediated mechanical cues in the ECM were first addressed in epithelial cell cultures [117]. Mammary gland epithelial cells (MECs) cultured on soft collagen gels or recombinant basement membranes resulted in the formation of spherical epithelial monolayers indicating that changes in the ECM have a profound effect on cell growth arrest, polarization, and ultimately differentiation [118,119]. It is suggested that various changes in the ECM can remarkably disrupt the spherical structure due to reductions in cell-cell junctions and depolarization that can contribute to enhanced cell proliferation and eventual migration [120]. The *Hippo* signal core components have an additional role in physical signals that affect the ECM and pathophysiological behavior in cell fate decisions [121]. *YAP/TAZ* components relay mechanical cues that influence the cellular microenvironment such as ECM stiffness, cell adhesion, and polarity [66]. *YAP/TAZ* controls organ size in several tissues during embryonic development through the progenitor’s amplification [122,123,124]. Proof of concept studies were performed showing crosstalk between the *Hippo* pathway and *Notch* signaling in the regulation of *YAP/TAZ*-mediated mechanical control of stem cell fate decisions [125]. *YAP/TAZ* acts as a mechanical signal transducer to maintain an undifferentiated state and preserves the different biomechanical signals, such as cell adhesion and apicobasal polarity. In contrast, *YAP/TAZ* inactivation in cells results in low mechanical signals which is instrumental in the loss of proliferation and differentiation. Overall, although *YAP/TAZ* regulation is not fully understood; observations to date indicate that *YAP* activity changes with respect to matrix stiffness and cell differentiation (Figure 5).

In the setting of metastatic disease, cancer stem cells (CSCs) associate with stromal and mesenchymal cells to maintain stable secondary tumor architecture in cancer metastasis [123]. Once metastatic cancer cells are stimulated to grow in secondary sites, *Hippo* signaling can be influenced by ECM components. Subsequently, nuclear *YAP* activity increases leading to the synthesis of growth factors resulting in cell proliferation and tumor growth [121,123]. The influence of ECM on *Hippo* signaling was shown in epithelial cells cultured on rigid compared to soft matrices with different matrix concentrations associating with the regulation of hormonal secretion (stiff ECM led to high secretion of progesterone and androgen, whereas soft ECM decreased steroidogenic hormones) [126,127]. Altogether, *Hippo* signaling molecules not only play a role in biochemical signals but are also involved as mechanotransducers to facilitate cancer metastasis. Overall, biomechanical cues such as stiffness and solids affect cancer progression and metastasis. Less rigidity or softer substrate is more permissive to cancer growth, cellular differentiation, steroid production, and metastasis. Understanding *Hippo* signaling during cancer progression has far-reaching implications for the identification of novel targetable mechanisms for better treatment options for metastatic cancers.

## 6. Future Directions

In recent years, investigations into the role of the *Hippo* pathway in cancer development and metastasis have increased at a remarkable pace because of the many advantages of gene identification and characterization in cancer research. Future work remains to identify pathway components in different tissues using various study models. A deeper mechanistic understanding is needed for crucial steps in the pathway, such as how the activity upstream signals influence *Hippo* molecules, how the levels of *LATS* protein phosphorylation complex is controlled, and what stimulates *Hippo* signaling upstream and downstream responsive elements. Attention to these mechanistic insights requires substantial investigations using in-vitro and in-vivo biochemical approaches. Factors enabling the dissection of essential and non-essential forms of pathway components would be valuable. Many upstream factors influence the remarkable features of the *Hippo* pathway with the primary challenge to understanding how the *Hippo* pathway crosstalk’s with other growth control pathways. For instance, a better understanding is needed concerning the role *YAP/TAZ* crosstalk with *Notch, Wnt*, and *Hedgehog* pathways to regulate mechanical stem cell fate decisions. A potential striking feature of the *Hippo* signal is the potential diversity in transcriptional responses and the ability of *YAP* transcriptionally coactivating with different DNA-binding partners. Still, to date, most research is focused on a limited set of molecules, downstream target genes, and the broad spectrum of cell/organ types. The diversity of transcriptional responses to *YAP* and post-translational modifications of *Hippo* core component activity remains an open question. Table 2 outlines the main mechanisms of the *Hippo* pathway, highlighting both established insights and areas that still need elucidation.

However, dysregulated *YAP/TAZ* activity in cancer has prompted the exploration of targeted therapies that can either inhibit or activate this signaling cascade to combat tumor growth. Recent studies have identified several therapeutic agents that can inhibit the transcriptional interaction between *YAP* and *TAZ*, leading to tumor growth inhibition [128]. Moreover, compounds like carbenoxolone may also inhibit the *Hippo* pathway, indicating that a dual role depending on their context of use, targeting upstream regulators such as *MST1/2* kinases, has emerged as a promising strategy to restore *Hippo* signaling and promote tumor suppression [129]. As our understanding of the *Hippo* pathway advances, new treatment modalities are expected to emerge, offering hope for more effective cancer therapies. Some of the therapeutic drugs are still under clinical stages (Table 3).

## 7. Conclusions

In solid tumor development, the maturation and metastasis of cancer cells are critical processes. Cancer progression and growth begin with CSCs, fueled by cytokines, mesenchymal cells, and stromal cells in cancer niches. Primary cancerous niches involve different stages of cancer development that are influenced by signaling molecules and pathways leading to epithelial cell proliferation and differentiation to support tumor growth and metastasis. The *Hippo* pathway molecules are a class of signaling molecules that can act as modulators of cancer metastasis with changes in activation stimulating a series of events involved in cancer progression. Contributing network molecules (e.g., *Hippo*, *Warts*, *Mats*, and the transcriptional coactivators) were first identified in the Drosophila. The *Hippo* cascade has highly conserved core elements involving two kinases, *MST1/2* and *LATS1/2* (related to *Drosophila’s Hippo* and *Warts*, respectively) which are potential regulators to maintain tissue homeostasis across the species. In mice, ovarian cancer development is regulated through *YAP, TAZ, MST1/2, SAV1*, and *LATS1/2 MST1/2* kinases. Murine studies suggested that due to the dysregulation of the *Hippo* signal, the transcriptional activity of *YAP* is significantly affected leading to aberrant actin polymerization, tumor growth, and metastasis. Further, activated *LATS1/2* phosphorylates *YAP* and *TAZ* to inhibit their nuclear translocation, resulting in *YAP/TAZ* transcriptional control. As detailed in this review, *Hippo* dysregulation plays a crucial role in several aspects of cancer progression and metastasis, while *YAP/TAZ* represent a potential therapeutic target in controlling the development and metastasis of gynecological cancers.

## Figures and Tables

**Table 1:** Core proteins of the *Hippo* Pathway.

Protein	Function	Role in *Hippo* Pathway
*MST1/2*	Serine/threonine kinases	Phosphorylates and activates *LATS1/2*
*LATS1/2*	Serine/threonine kinases	Phosphorylates *YAP/TAZ*, leading to their inactivation
*YAP*	Transcriptional co-activator	Promotes cell proliferation when unphosphorylated
*TAZ*	Transcriptional co-activator	Similar function to *YAP*
*SAV1*	Scaffold protein	Assists *MST1/2* in activating *LATS1/2*
*TEAD*	Transcription factor	Binds *YAP/TAZ* to promote expression of target genes
*NF2(Merlin)*	Tumor suppressor protein	Activates the *MST1/2* kinase cascade

**Table 2:** Understanding Achieved and Aspects Still to Explore in the *Hippo* Pathway.

Mechanism	Known	Remains to be Elucidated	Reference
Core Components	The pathway is primarily regulated by *MST1/2*, *LATS1/2*, *YAP*, and *TAZ*. *MST1/2* activates LATS1/2, leading to *YAP/TAZ* phosphorylates and inactivation.	The precise mechanisms regulating *MST* and *LATS* activity, including upstream signals and feedback loops, require further exploration.	[4,23,33,34,123]
*YAP/TAZ* Regulation	*YAP* and *TAZ* act as transcriptional co-activators that promote cell growth and survival when unphosphorylated and translocated to the nucleus.	The full spectrum of *YAP/TAZ* target genes and their contributions to tumorigenesis and other diseases requires further investigation.	[20,43,66,85]
Cross-Talk with Other Pathways	The Hippo pathway interacts with *Wnt*, *TGF-β*, and *EGFR* signaling, influencing various cellular processes like proliferation and differentiation.	The details of how these pathways integrate at a molecular level and how they affect *YAP/TAZ* function in different contexts are not fully understood.	[12,66,123]
Role in Cancer	Dysregulation of the *Hippo* pathway is implicated in many cancers, often through overexpression of *YAP/TAZ*.	The mechanisms by which *YAP/TAZ* contribute to specific cancer types and the role of the tumor microenvironment in modulating *Hippo* signaling need to be elucidated.	[15,65,66,72,85]
Developmental Functions	The *Hippo* pathway is crucial for regulating organ size and stem cell maintenance during development.	The exact roles of the *Hippo* pathway in various developmental stages and its impact on tissue homeostasis remain to be clarified.	[66,123]
Therapeutic Potential	Targeting the *Hippo* pathway has been proposed as a therapeutic strategy for cancer treatment.		[7,23,94,123]

**Table 3:** Some therapeutic agents target the core elements of the *Hippo* pathway.

S.No.	Drug/Agent	Description	Clinical Trials
1	Verteporfin	Photosensitizer, Inhibits *YAP/TAZ* interaction	NCT02512755
2	CA3	Promoting apoptosis	Pre-clinical
3	HSF1	Inhibiting *YAP* activity	Pre-clinical
4	SBP-3264 XMU-MP-1	Targeting *MST/LATS* kinases	Pre-clinical
5	VT3989 (Vivace Therapeutics)	*TEAD* palmitoylation inhibition	NCT04665206
6	IK-930 (Ikena Oncology)	*TEAD* palmitoylation inhibition	NCT05228015
7	ION537	Anti-*YAP* activity	NCT04659096
8	IAG933	*YAP/TAZ* fusions	NCT06566079
9	zoledronate	*YAP/TAZ*	NCT02347163

## Data Availability

No new data were created or analyzed in this study.
